# Medical Items and News of Interest to the Physician

**Published:** 1881-04

**Authors:** 


### Medical Items and News,

### For the Use of Physicians.

CULLED FROM THE STANDARD MEDICAL JOURNALS OF THE WORLD.

**Syphilitic Neuralgia.**

Dr. Mauriac employs the following formula:

℞

Pulvis iodoformi, ℈ i;

Ext. et pulvis gentianæ, q. s.

Fiat in pil. no. xx.—M.

Two or three to be taken daily.

**Styptic Collodion.**

The following is used in the hospitals of New York:

℞

Tannin, ℥ ij.
Absolute alcohol, fl. ℥ ss.
Ether, fl. ℥ ijss.
Collodion, q. s. ad fl. ℥ xij.

**Cystitis.**

Dr. Deecke considers lactic acid the best of all acids. It has better and more durable results.

Lactic acid 1 gramme.
Water, sweetened, q. s.

Take three or four times a day. Lactic acid is found in the urine after three or four hours. It arrests safely the ammoniacal decomposition, dissolves the salts which abound and destroys the vegetable mscroscopical formations.—*From Jour. de Medicine in Ther. Gazette.*

**Diphtheria.**

(*Jour. de Med. et de Chir. Pratiques*, June, 1880.)—M. Vidal recommends the topical use of tartaric acid in diphtheria. In his opinion it is necessary to make use of topical agents against the false membrane, for it has a great tendency to spread by a sort of auto-inoculation, comparable to what occurs in certain cutaneous affections. The following is the formula he gave to the Therapeutical Society as the one he uses:

℞

Tartaric acid, 10 parts by weight.
Glycerine, 15 " " "
Mint-water, 25 " " "

The acid acts upon the false membrane, converting it into a gelatinous mass, and favors its expulsion. The parts should be swabbed about every three hours, following the application after a short time, with one of lemon juice.

**Esmarck's Caustic Powder.**

Arsenious acid, 15 grains.
Sulphate of morphia, 15 grains.
Calomel, 2 drachms.
Powdered gum arabic, 12 drachms.

Mix. This is sometimes called *painles caustic powder*, although the propriety of the name is doubted by some.

**Burns.**

Patient badly burnt from the ignition of the vapor of ether; fifteen minutes afterward the wound was freely strewn with bicarbonate of soda pulverized—the severe pain ceased almost immediately—after one and a half hours the soda was removed, redness of the skin and tenderness were only present where at certain places the soda had crumbled and broken off. At these places, the next day, bullæ had formed, otherwise the skin was normal.—D. M. Hezenstein in *Medical Gazette.*

**Remedies for Chilblains.**

The following formula for Dr. Valentine Mott's Remedy is given in the *Proceedings* of the Medical Society of the County of Kings:

℞

Beef's gall, 4 ounces.

Ol. terebinth, 4 ounces.

Spts. vini rect., 90 per cent., ½ ounce.

Tinct. opii, 1 ounce.

Another formula for the same affection is:

℞

Beef brine, 1 pint.
Potassæ nitratis, 2 drachms.
Aquæ ammoniæ, 3 ounces.

**Syphilis.**

After dwelling upon the importance of exhausting every conceivable means of diagnosis, Sigmund, as the result of his long experience, advises removing the initial lesion (if the case be seen very early) with knife, cautery, or caustic, followed by neat dry dressings. After this he advises deferring constitutional treatment, except hygienic, until the cutaneous manifestations appear. When this arrives, he uses for the lighter forms the iodine preparations; for graver forms with defective nutrition and strength, palpably due to syphilis alone, or widespread pustular, papular, or squamous eruptions, mercury. But this must never be pushed to salivation. For the gravest tertiary forms he recommends mercury and iodides alternately.—*Neuere Behandlungsweise der Syphilis.*

**Uterine Engorgement.**

℞

Glycerine, 150 grammes.
Acid tannic, 40 grammes.
Tr. iodine, 40 grammes.

M. Let it dissolve and filter. A tampon saturated with this medicine is applied to the neck of the womb, and kept in place for six or eight hours, and then repeated. The results are beneficial.—*Review de Medicale, in Ther. Gazette.*

---

**Urethritis.**

Dr. J. P. Zeitlin treated fourteen cases of uncomplicated urethritis with chlorate of potash as described by Bochmann (Kars) *i. e.* three grams daily, in all of which it was followed by favorable results. Even after a few doses, the discharge became thinner and pain and erections subsided; there were no bad effects of the drug. He attributes its action to its rapid excretion by the kidneys in unchanged form and hence as acting locally upon the urethral mucus membrane.—*Cent. fur Chir.*

---

**Battey Solution for Eczema.**

℞

Iodini cryst., ½ ounce.

Acid carbol. cryst, 1 ounce.

Combine the two by gentle heat.

Dr. Bellamy, of Wilmington, N. C., states, in the *North Carolina Medical Journal*, December, that the above formula has given him more satisfaction in the management of those intractable forms of skin disease characterized by intolerable itching, and more particularly in eczema marginatum, than any other parasiticide. [This seems to be rather a strong solution (?) Use with care.—ED. BISTOURY.]

---

**Tonic Glycerine.**

There are many patients to whom cod liver oil cannot be administered, as it destroys their appetite, and produces dyspepsia. Dr. Larmande highly recommends "tonic glycerine" as a substitute for the oil in such cases. This is made by adding 30 drops of tincture of iodine, and 5 grains of iodide of potassium, to 9½ ounces (by weight) of glycerine. A tablespoonful should be taken a quarter of an hour before each meal. This generally restores the appetite, and removes constipation if it be present. For children or delicate persons, one-sixth part of the glycerine may be omitted, and replaced by syrup of raspberries.—*Lyon Medical*, April, 25, 1880; *The Glasgow Med. Jour.*

**Soda Mint.**

This excellent antacid for the stomach is made as follows:

Bicarbonate of Soda, 4 drachms.
Aromatic spirit of ammonia, 1 ounce.
Spearmint or peppermint water, 16 ounces.

Mix. Dose, from a dessertspoonful to a tablespoonful for adults.

---

**Gleet.**

Having hit upon a remedy with which I am having good success in the treatment of this disease, I deem it my duty to place it before the readers of your valuable journal. It is the following:

℞

Zinci. Sulph. 12 grs.
Tinct. Iodine, 10 drops.
Water 8 ounces.

M. Sig. Inject four times a day.

Also—

℞

Ext. Uva Ursi fld. 3 ounces.
Ext. Pareira brav. 1 ounce.
Ext. Cascara sag. 2 ounces.
Syr. Orange peel, 2 ounces.
Water q. s. ad. 8 ounces.

M. Sig. Teaspoonful three times a day before meals.

I consider this an invaluable remedy in obstinate cases.—*S. L. Blake, M. D., in Medical Brief.*

---

**Dropsy.**

Sarah M., æt. 54, dark hair, fair, and delicate skin, consulted me January 23, 1879. About fifteen months ago she began to have swelling of the feet and legs at night; this increased gradually without any swelling of the face or eyelids; but the lower extremities, up to the hips, became gradually larger, and about two months prior to my seeing her she observed that her abdomen was enlarging rapidly. As the ascites increassd she began to feel very ill; loss of appetite and general debility; no renal trouble until a short time before coming to me; then the urine began to decrease daily. Urine did not yield albumen on the application of either heat or nitric acid. I put her on fluid extract haircap moss, two drachms in a cupful of water, four times a day, and increased gradually to eight times during the day. Kept the bowels open with antibilious physic. Gradually the swelling diminished, and at the end of two months I feel sure she is perfectly cured.—*T. A. McKinney, M. D., in Medical Brief.*

**Treatment of Barber's Itch.**

Brame recommends the following treatment: Shave off the hairs, or cut them very short; then apply once or twice a week, an ointment composed of:

℞

Prepared chalk, 10 parts.
Coal tar, 1 to 4 parts.
Glycerine, 5 parts.
Simple cerate, 50 parts.

---

**Fever and Ague.**

Dr. Lo Feren contributes an article in the revisto de Medicina Cirugia Practicus, Madrid, on this disease. In this locality (Guadalajara) one of those epidemics of fever and ague reigned, which disgust medical men, by the severity of the fever and the uselessness of quinine in all forms, and other specifics for the disease. After vainly trying these medicines I commenced with the telerana; it gave excellent results in doses of two and a half grains in pills, taking twenty grains the first day, eight the second, four the third day, and two grains the fourth day.

During the past three years we have found no case of intermittent fever which did not yield to the above treatment.—*Rev. Med. Madrid in Ther. Gaz.*

---

**Abdominal Typhus by the Douche.**

Dr. Marcowitz, of Bucharest, has arrived at the following conclusions in regard to the treatment of abdominal and petechial typhus by the douche: 1. The douche allows of the regular and methodical treatment by cold applications of twelve or fifteen severe cases of typhus (two to seven or eight douches for each patient,) without occupying more than two or three hours a day. 2. The patient is more effectually roused by the douche than by the cold bath; this is probably explained by the fact that, independently of the subtraction of heat, the shock acts in a reflex manner upon the heat-centres and prevents their paralysis for a period which may extend over some hours. 3. As contra-indications, Dr. Marcowitz agrees with Liebermeister, that only intestinal hæmorrhage, or paralysis of the heart to a great extent with a small and weak pulse, are sufficient to prohibit absolutely the administration of the douche. 4. Pulmonary engorgement so long as it does not depend directly on paralysis of the heart is rather an indication than a contra-indication for this method of treatment.—*Arch. Geu. de Med.*

**Herpes Circinata, Herpes Zoster, and Herpes of the Prepuce.**

Dissolve one drachm of iodoform in one-half ounce of the oil of eucalyptus, and paint the diseased surface with this solution. Two or three applications will usually effect a cure.

---

**Leucorrhœa, or Vaginal Catarrh, and its Treatment.**

Leucorrhœa is a catarrh of the vagina, and sometimes the cervix. It is not a disease *per se*, but a symptom, and is as offensive to the patient as it is difficult to treat. It is my opinion that it is a species of scrofula, when not due to mechanical causes, and the practical men in France agree with me. The treatment I have tried and seen tried here, and which has produced the most beneficial result, is cod-liver oil, iron, and quinia, with a soluble capsule or suppository, such as is made by Park, Davis & Co. These soluble pessaries are superior to the usual ones made in drug stores, because one-half the druggists do not make them right. When leucorrhœa complicates any malposition of the uterus, pessaries are indicated; should be oiled before introduction and frequently removed, lest inflammation results.—*L. G. Doane, M. D., in Medical Brief.*

---

**Gonorrhœa and Vaginitis.**

Gurjun balsam, which is extracted from various dicotoledonous trees, is turbid and of a brown color, bitter, smelling like copaiba, but with a less unpleasant odor, while it is less acrid and less expensive than this drug. It is also more readily tolerated. The balsam is administered in capsules or in the form of an emulsion in mucilage. In the latter form Vidal gives four grams a day immediately before meals. In large doses of ten to twelve grams it produces vomiting and diarrhœa. It can be prescribed at the beginning of a gonorrhœa, which it cures in fifteen to twenty days. It is better, however, to employ it when the inflammatory stage is over; but it is also very useful in gleet. In the female it is used as a local application. The vagina being first washed out with warm water, a plug of cotton wool soaked in a liniment of equal parts of balsam and lime water and applied by means of the speculum, and the plug is then covered with a second one of dry wool, the dressing being renewed daily. This proceeding is attended with a slight smarting, which disappears after the third application.—*Bouchut's Ann. de Therapeutique.*

**A Menstruum for Salicylic Acid.**

In the *Louisville Medical News*, Dr. Springer states that salicylic acid is readily soluble in effervescing vichy or seltzer water, the former, from containing an excess of alkaline carbonates, being preferable. The acid is put into a tumbler first and mixed thoroughly with a small quantity of water, to prevent its floating, and the glass is then filled with the effervescing water and the liquid drunk off. When perfectly dissolved it is said to have a very pleasant, exhilerating, pungent, and sweetish taste.

---

**Castor Oil.**

A palatable and agreeable method of taking castor oil is suggested by the Boston Medical and Surgical Journal, viz., make an emulsion containing

Castor oil, ℥j.

Tinct. cardamon comp., ʒ iv.

Ol. gaultheriæ, gtt. iv.

Pulv. acaciæ et pulv. sacchari alb., āā ʒ ij.

Aq. cinnamon, q. s., ℥iv.

Mix.

A German method especially agreeable to children is to make the confection of castor oil into a paste, with either about three parts of coarsely granulated sugar or two parts of comp. liquorice powder; and flavored with powdered cinnamon or grated lemon peel.—*Boston Med. and Sur. Journal.*

---

**Microscopic Examination of Blood.**

The characteristic distinctions of human blood and those of the blood of other animals are thus set forth in No. 2 of *L'Orosi* by Dr. Vincenzo Peset y Cervera, and if other physiologists will take the pains to verify his work, they may play a very important part in medical jurisprudence. When blood is mixed with a little bile, small crystals not over 0.003 of a metre in size are formed; but these crystals, the Doctor says, will show whence the blood had come. If from man, the crystals will be rectangular prisms; if from the horse, they will be cubes; if from the ox, they will be rhombohedrons; if from the sheep, they will be rhombohedric tablets; if from the dog, they will be rectangular prisms, closely resembling the human forms; if from the rabbit, they will be tetrahedrons; if from the squirrel, they will be hexagonal tablets; if from the mouse, they will be octahedrons; and if from common poultry, they will be cubes more or less perfect. There would seem to be room for more accurate research in this direction.—[We should say so.—ED. BISTOURY.]

**Argyria Following the Frequent Pharyngeal Application of Nitrate of Silver.**

A woman, aged forty-six years, noticed a bluish discoloration of the entire cutaneous surface, following repeated pharyngeal cauterizations with the silver nitrate stick. Similar cases have been recorded, one by Krishaber, and a second in the *Gazzetta Medica Italiana* (1862). The absorption of the silver salt takes place in part from the mucous surface of the cauterized portion, but principally from the intestinal surface, the products of cauterization being conveyed to the alimentary canal.—*Archives Med. Belges.*

---

**Vapors for Inhalation.**

The following are selected by the *Monthly Magazine of Pharmacy* from the formulæ used at the Hospital for Diseases of the Throat in London:

VAPOR CARYOPHYLLI.

Oil of cloves, 30 minims.
Light carbonate of magnesio, 15 grains.
Water, 3 ounces.

VAPOR CASSIÆ.

Oil of cassia, 20 minims.
Light carbonate of magnesia, 10 grains.
Water, 3 ounces.

VAPOR CINNAMONI.

Oil of cinnamon, 20 minims.
Light carbonate of magnesia, 10 grains.
Water, 3 ounces.

VAPOR CREOSOTI.

Beechwood creosote, 3 drachms.
Glycerine, 3 drachms.
Water, 3 ounces.

VAPOR CUBEBÆ.

Oil of cubebs, 2 drachms.
Light carbonate of magnesia, 60 grains.
Water, 3 ounces.
Useful in laryngorrhœa.

VAPOR CUBEBÆ C. LIMONE.

Oil of cubebs, 1½ drachms.

Oil of lemon, ½ drachm.

Light carbonate of magnesia, 60 grains.

Water, 3 ounces.

The oil of lemon is added to mask the disagreeable odor of the cubebs.

A teaspoonful to be added to a pint of water at the desired temperature, 150 F., and an additional teaspoonful to be added every five minutes during the time that the inhalation is used. Not more than three teaspoonfuls to be used on any single occasion.

**Danger of Injections of Tincture of Iron in Nævus.**

A correspondent of the *British Medical Journal* says: "A child, ten months old, was brought with a small nævus on the right side of its head. I injected five drops of tincture of perchloride of iron with two drops of water. In about a minute the child's face was of a peculiar pea-green color, with black stripes—the veins. The tip of the syringe had entered a small vein. After four hours' hard work, and with the greatest care, the child's life was saved. Since then I have never used injections for the cure of nævi."

---

**Cheken.**

Dr. Henry Von Dessauer, of Valparaiso, has used cheken for some years in the treatment of a number of complaints. Thus as an inhalation he uses it in dipththeria, laryngitis, bronchitis, as an injection in certain affections of the mucus membrane, as gonorrhœa, leucorrhœa, cystitis, etc., when given internally, in the form of syrup or liquid extract, it is said to aid digestion, allay cough, facilitate expectoration, and stimulate the kidneys to action. It is also an astringent, and is found to be of especial service in threatened hemoptysis. Dr. von Dessauer used it with marked success in more than one hundred cases of bronchitis and phthisis. For many years he was physician to a large convent school, many of the inmates of which suffered from consumption, and hemoptysis being of constant occurrence. During the two and a half years he gave cheken in this establishment he had not a single death from phthisis, there were no fresh cases of hemoptysis, and many of the patients who had had repeated attacks of bleeding from the lungs recovered and gained flesh and strength in a very marked manner.—*Louisville Medical News.*

---

**Chest Affections.**

Dr. Reuss, of Paris, has, according to the Glasgow Medical Journal employed creasote in the treatment of phthisis with apparent benefit. He prescribes it in lozenges (dragees,) the formula for each being:—

℞

Pure balsam of tolu, 20 per cent. (3 grs.)
Pure beech cresote, 5 per cent. (¾gr.)
Excipient, q. s.

Sig. Two of these for a dose; given at first night and morning, and gradually increased, sometimes up to ten lozenges in a day.—*Medical and Surgical Reporter.*

**Tonga.**

Tonga is a new remedy, introduced from the Fijee Islands, and consists of a mixture of bark, leaves and fibres, which, according to Holmes, are probably derived from Raphisdophora vitiensis. The remedy is recommended against neuralgia, and is said to contain a volatile alkaloid, Tongina. A brown liquid extract of the drug has been made in London.—*American Journal of Pharmacy.*

---

**Chlorate of Potassium and the Increase of Nephritis.**

In his work on diphtheria, Dr. A. Jacobi says that chlorate of potassium or soduium is used more probably than any other drug. It is used domestically, and is then not weighed out, but taken indiscriminately. Acute nephritis, probably, and chronic nephritis, certainly, is oftener met with now than it was formerly. Dr. A. Jacobi is inclined to think that the chlorates are partly to blame for this increase in nephritis. Such a view, says the *Medical Record*, is rather a transcendental one.

---

**Purgatives in Phthisis.**

M. Ferrand, in his clinical lectures upon phthisis, recommends that the following mixture should be administered in a cup of tea when it is necessary to relieve constipation in phthisical patients (*Le Progres Medical;*) Calcined magnesia, 2 to 4 grammes; manca in tears, 30 to 40 grammes. If this laxative has to be taken often, it may be converted into an electuary by the addition of a little honey. In this form a large teaspoonful is to be taken every morning: Manna in tears, 30 grammes; calcined magnesia, 4 grammes; white honey, 30 grammes.—*Practitioner.*

---

**Diarrhœa of Infants.**

Dr. Demme, of Berne, recommends the following:

℞

Cognac, 2 to 5 grammes.
Creosote, 1 centigramme.
Drops of tar, 1 to 5 grammes.
Distilled water, 50 grammes.

M. The whole to be taken in twenty-four hours. Among very young infants, the quantity of alcohol can be increased to five grains. The object of this potion is to stimulate nutrition and to prevent the formation of microspores, which encumber the intestinal glands.—*Therapeutic Gazette.*

**Itch.**

℞

Balsam of Peru, ℥j;
Benzoic acid, grs. 110;
Oil of cloves, gtt 40;
Alcohol, ʒijss;
Simple cerate, ℥vij.

Dissolve the essential oil and the benzoic acid in the alcohol, and mix them with the cerate. Lastly, add the balsam of Peru. It is said to effect a cure in twenty-four hours.—*Med. and Surg. Reporter.*

---

**Balsam of Peru in Pruritus.**

In a communication to the *Deutsche Med. Wochen.*, Dr. Auerbach, of Berlin, states that having, in common with so many other practitioners, found the balsam of Peru a most valuable remedy in itch, he has for some time past used it in the treatment of pruritus with the greatest success. After the first rubbing into the part affected great relief is obtained, and in a few days a cure results. He relates a very obstinate case which, after resisting all kinds of treatment for years, was speedily cured by the balsam.

---

**Vermifuge.**

Dr. A. S. Sweet, of Southold, L. I., in a communication to the *Medical Brief*, recommends the following mixture:

Santonin, 16 grains.
Fluid extract of pink root, 160 drops.
Simple syrup to complete, 2 ounces.

Mix. Sig. Teaspoonful morning and night.

It was given about equally between four children of one family and one of the neighbor's children. The result was the expulsion of sixty-seven long worms. He adds: "As having a possible bearing upon the question whether worms cause any special symptoms by their presence in the intestines, allow me to say that the child for which the vermifuge was particularly desired, had, previous to taking it, several attacks of convulsions. They ceased with the expulsion of the worms.

---

**Cystitis.**

Dr. W. H. Smith, in *The Physician and Surgeon*, contributes an article upon cystitis, the sum and substance of which is as follows: Describing a case he says: "He was complaining of intense pain in the region of the bladder, which was aggravated by passing water and by motion. These pains were of a lancinating character and occasionally extended up the loins. His urine was also pale, watery and greatly in excess, amounting to one and a half gallons per day, and contained considerable albumen and a quantity of pus. He had also at times passed blood. These facts awakened a suspicion of stone or a growth within the bladder, but an examination failing to reveal either of these, the diagnosis was arrived at of cystitis with diabetes insipidus. The presence of the albumen was explained as being due to the cystitis and the polyuria had undoubtedly been caused by the abuse of diuretics."

The next day the patient was put upon a milk diet and the following prescription:

℞

Extracti eucalypti globuli fluidi, ʒij.

Extracti belladonnæ fluidi, ʒss.

Acidi benzoici, ʒj.

Syrupi tolutani q. s. ad. ℥vj.

M. Sig. A dessertspoonful in half a glass of milk every three hours.

The importance of rest in the horizontal position was enjoined and after one day the amount of medicine was reduced to a dose every night. This had an excellent effect. The pain diminished rapidly, and the drug each evening appeared to induce a good night's rest.

---

**The Treatment of Asthma.**

I wish to draw attention to a method of treatment of asthma, described by Dr. R. B. Faulkner, in the New York Medical Record for September 25th. His plan is to paint a strip of iodine over the course of the pneumogastric nerves in the neck. He gives three cases of pure spasmodic asthma, which were relieved of their attacks by this means, after having resisted every other remedy of which he could think.

I have tried it in several cases; but all except one, were not pure spasmodic asthma, and in this case the benefit was not marked. R. S., aged 67, had asthmatic attacks at 2 A. M. every morning; he had had them for the last nine days; never had them before. He had lived in the same house for the last thirty years; had never had gout. There were no physical signs of disease in the thorax; no albumen in the urine. I ordered iodine pigment, as above. He returned in a week, expressing the liveliest sense of the relief he had received. In the other cases, some seemed relieved; but the benefit was not so decided.—*Robert Saundby, M. D., M. R. C. P., in British Med. Journal.*

**Compound Camphor Powder.**

Dr. D. S. Clark, of Rockford, Ills., recommends as follows:

The following formula, when carefully prepared, is a very pleasant mode of administering morphine. The amount of morphine can be varied, so as to make 10 grs. equal in soporific effect to 10 grs. of Dover's powder:

℞

Morphiæ sulph, gr. xiv.

Pulv. camphoræ.

Pulv. rad. glycyrrhizæ.

Soda bicarb, āā ʒ iv. M.

---

**Salicylic Acid for Bee Stings.**

An Austrian paper recommends the following treatment: First remove the sting as quickly as possible with a forceps or by scratching with a finger, but never with the thumb and forefinger, because this squeezes more of the poison into the wound. Next squeeze the wound until a drop of blood comes out, and rub the place as large as a dollar with an aqueous or dilute alcoholic solution of salicylic acid. The effect is still better by injecting the salicylic acid into the wound with the hypodermic syringe. After this the spot is painted with collodion to keep out the air. A sting treated thus causes little or no pain, slight inflammation and swelling, and is not followed by nettle fever or lameness in the most sensitive and nervous individuals.

---

**Nitro-Glycerine.**

In *The British Medical Journal* Mr. A. W. Mayo Robson confirms the value of nitro-glycerine in cases of migraine, angina pectoris, and asthma, one severe case of this latter disease being most remarkably cured by the drug. Soon after the powers of nitro-glycerine were brought before the profession by Mr. Field in the *Medical Times and Gazette*, 1858 and 1859, the marvelous powers which the drug possesses of relieving the spasm of asthma were fnlly tested by the reporter among patients living in the malarious climate of Java, were the disease is very common. Frequently the tongue of the patient was touched with the stopper of a bottle containing a 5 per cent. solution, when the paroxysm was so severe as to cause consternation to all around, and in less than a minute there was a great calm; the patient, especially if a Chinaman or Malay, believing himself to be the subject of a miracle.

**Pharyngitis.**

In chronic pharyngitis, where the blood vessels of the pharynx are enlarged and tortuous and the secretion moderate, the following is recommended:—

℞

Ergotine, gr. xx

Tinct. iodine; f. ʒ j

Glycerine, f. ℥j.

M. Sig. Apply to the pharynx freely, twice daily, with a camel's hair brush.—*Med. and Surg. Reporter.*

---

**Heart Disease.**

Dr. Potain (Bull. Gen. de Therap., vol, ii., 1880, p, 232) says that milk diet is particularly efficacious in secondary effections of the heart, hypertrophy or simple dilatation of renal or gastric origin. This regimen acts on the stomach or kidneys by affording complete repose. It should be continued for a long period. It is useful also in simple reflex palpitation of gastric origin. In order to be efficacious, the milk regimen should be well borne by the stomach. Three quarts daily appears to be the proper average amount to be ingested.—*Med. and Surg. Reporter.*

---

**Formula for Hypodermic Use in Syphilis.**

Dr. Yvon (Bull. Gen. de Therap., 1880, vol. ii. p. 217) recommends the following as not exciting local iritation, not coagulating albumen, and as easily absorbed:

℞

Hydrarg, biniodid., } āā gr. xv;

Potassii iodidi, }

Sodii phosphat. (tribasic,) ℈iss;

Aquæ destillat., f ʒ xij.—M.

Six minims of this solution will contain one-eighth of a grain of the mercurial salt.—*Med. and Surg. Reporter.*

---

**Small Pox Pitting.**

The face is covered by a thin mask coated on one of its sides with the following preparation:

Phenic acid, 4 to 10 grammes.
Olive oil, 40 grammes.
Chalk, powdered, 60 grammes.

Or this:

Thymol, 2 grammes.
Linseed oil, 40 grammes.
Chalk, powdered, 60 grammes.

The mask covered with one or the other of these liniments is renewed every twelve hours. —*Le Scalpel, in Ther. Gaz.*

**Whooping Cough.**

The good effects of bromide of potassium in the treatment of whooping cough are well known to all practitioners. According to Dr. Wintreben, in La France Medicale, the action of this remedy may be made still more efficacious by bringing it in contact with the mucus membrane of the air passages in the form of spray. The author habitually uses a solution of bromide of potassium, one in twenty, and repeats the application of the spray for one minute after each fit of coughing, when the mucus membrane of the breath passages, free from the mucus which usually covers it, is accessible to the action of the remedy.—*Med. and Surg. Reporter.*

---

**Strychnia as a Physiological Antidote to Alcohol.**

Dr. Luton, in the *Bulletin de Therapeutique*, claims that by frequent experiment he has demonstrated that strychnia is the best physiological antidote in cases of chronic alcoholism. He has used hypodermic injections of the sulphate of strychnia in delirium tremens with markedly favorable results, relieving tetanic rigidity and quieting delirium.

This tends to confirm the truth of the stories about strychnia eating in California, which were noticed in *The Druggists' Circular* some five years since. It will be remembered that, according to reports made in evident good faith by our correspondents, the practice was resorted to by hard drinkers, and had become almost a fine art.

---

**Diphtheria.**

Pilocarpiæ is recommended on the strength of the assertions of Dr. Guttman, of Berlin. His prescriptions were, however, not quoted. They are as follows, he combining pepsine with the alkaloid, in order to combat the gastric catarrh present:

℞
Pilocarpiæ muriat, gm. 0·02—0·04;
Pepsinæ, gm. 0·6—0·8;
Acidi hydrochlor, gtt. ij;
Aquæ dest, gm. 80·0;
M. Sig. A teaspoonful hourly, for children.

For adults:

℞
Pilocarpiæ muriat, gm. 0·03—0·05.
Pepsinæ, gm. 2·0.
Acidi hydrochlor, gtt. iij.
Aquæ dest, gm. 240·0.
M. Sig. Hourly, a tablespoonful.

Dr. Rothe, of Altenburg, says (*Med. Cent. Zeitung*, Nov. 6) that four years ago he tried jaborandi in two cases, but both died, and he renounced the experiment. This throws some doubt on the value of Guttman's discovery.

The price of pilcarpiæ is high; it sells in the Eastern cities at thirty-five cents per grain, which makes it almost prohibitive in many cases.

---

**Chronic Bronchitis.**

Essence of Scotch snuff and paregoric, one tablespoonful to 240 grammes of hot water. This is put in an inhaler and frequently used. Most of the balsamic resins can be similarly used. Also

Crystalized phenic acid, 1 to 80 centigrammes.
Paregoric, 90 grammes.
M. One tablespoonful in 240 grammes of hot water.

The vapor of phenic acid and of camphorated opium calms the bronchial irritation and diminishes the frequency and abundance of expectoration.—*Translation in Ther. Gazette.*

---

**Iodide of Potassium in Cardiac Dyspnœa.**

Iodide of Potassium has been found by Professor See to work well in all cases of cardiac dyspnœa, particularly when this is connected with some structural lesion. It is equally useful in valvular lesions. No evil results can occur from its use, even if a mistake is made and the affection is asthmatic. The iodine liquifies the bronchial secretion. The dose is twenty grains a day, gradually increased to two or two and a half scruples. A good formula is:

℞

Potas. iod, ℨ vss.

Syr. aurantii cort, f. ℥ iv.

Sig. Two to four teaspoonfuls a day in a tumbler of water.

Patients suffering from heart disease are more tolerant of iodide of potassium than other patients. The contra-indications to its use are: 1, tendency to hemorrhage; 2, loss of flesh; 3, loss of strength; 4, loss of appetite. Opium may be added to prevent iodism. Another useful combination is digitalis with iodine, as one has a soothing influence on the dyspnœa by acting on the lungs, and the other increases the action of the heart and modifies the arterial tension. The following formula will be found to answer well:

℞

Potas. iod, ℨ ss.

Tinct. digitalis, f. ℥ ss.

Syr. acaciæ, f. ℥ iv.

Sig. Teaspoonful four times a day.

When digitalis is unsuitable, chloral may be substituted.

